# Clinical spectrum and prognosis of pathologically confirmed atypical tumefactive demyelinating lesions

**DOI:** 10.1038/s41598-023-34420-4

**Published:** 2023-05-13

**Authors:** Yajing Zhang, Ting Zhang, Xuebin Zhang, Xiaoling Yan, Jing Lei, Ran Liu, Yun Yang, Chao Zhang, Jun Zhang, Ying Zhang, Wei Yue

**Affiliations:** 1 Department of Neurology, Tianjin Huanhu Hospital, Jizhao Road 6, Tianjin, 300060 Jinnan China; 2 Department of Pathology, Tianjin Huanhu Hospital, Tianjin, China; 3 Imaging Department, Tianjin Huanhu Hospital, Tianjin, China

**Keywords:** Neurology, Oncology

## Abstract

To describe the clinical spectrum and prognosis of atypical tumefactive demyelinating lesions (TDLs), which were confirmed by pathology. A total of 11 patients were diagnosed with atypical TDLs confirmed by brain biopsy and surgery between January 2006 and December 2017. The clinical spectrum and prognosis in these patients were analyzed. The patients’ ages ranged from 29 to 62 years, with a mean age of 48.9 years; 72.7% were males. The Expanded Disability Status Scale (EDSS) of the patients with first onset was 2.36. Most of the patients started with limb numbness and weakness (45.5%) or alalia (27.2%). The mean time from symptom onset to biopsy or surgery was 12.9 days (3–30 days). Most of the patients had solitary lesions (72.7%), supratentorial lesions (90.9%, particularly predominant in the frontal, temporal, and parietal lobes), moderate edema (63.6%), mild mass effect (54.5%), and patchy lesions (54.5%). Among them, three patients were positive for myelin basic protein (MBP) and one patient was positive for myelin oligodendrocyte glycoprotein (MOG). The patients were followed up for an average of 6.9 years (2–14 years), and recurrent TDLs were observed in 2 patients. Except for the 2 patients who relapsed, only 1 of the 9 patients died; the other 8 patients improved or maintained the status quo (the EDSS scores were lower or unchanged). The patients did not have any serious nervous system injury at onset, and the main presentation included extremity weakness, headache or dizziness, and alalia. The most common form was patchy on MRI enhancement. Cerebrospinal fluid and demyelination test can be an indicator of TDLs, and seizures may be a poor prognostic indicator. Most atypical TDLs have monophasic courses and good outcomes. The effect of neurosurgery alone was good in our group, and the effect of surgery on atypical TDLs can be further studied.

## Introduction

Tumefactive demyelinating lesions (TDLs) can also be called “demyelinating pseudotumors”, “tumor-like demyelinating lesions (TDLs),” “tumefactive demyelinating lesions,” or “tumefactive multiple sclerosis lesions”. TDLs are tumors or lump-like white matter space-occupying lesions, generally larger than 2 cm^1^. TDLs generally have small edema and mass effects. It is difficult to differentiate TDLs from brain tumors, but most TDLs have a high degree of specificity, especially in radiographic features. Most of the TDLs showed incomplete annular enhancement, and the open side faced the cortex, with dilated veins centrally. MRI of TDLs are multiple lesions with high minimal apparent diffusion coefficient values^2^. TDLs require serial MRI scans, and some continuous evolutions will be found. However, a brain tumor will show stability in an MRI scan^3^. However, there are certain TDL lesions that are atypical on imaging and difficult to distinguish from tumors or the TDLs not responding well to immunotherapy, which are called atypical tumefactive demyelinating lesions.

The prevalence of TDLs has been reported to be 1–2/1000 for multiple sclerosis (MS)^1,4–7^. TDLs have been associated with multiple sclerosis (MS) and neuromyelitis optica spectrum disorders (NMODS). However, patients with TDLs could not fully meet the diagnostic criteria of MS and NMODS; therefore, TDLs may be an independent diagnosis^1,6,8,9^.Some reports about TDLs found that the conversion rate to MS or recurrence fluctuated between 10%^8^ and 70%^10^; this difference is huge. It is difficult to assess the prognosis of patients with TDLs. The possible risk factors of relapse include age, enhancement pattern, and the presence of other typical MS lesions^7,9^.

Some patients were not suspected of having TDLs before brain biopsy and neurosurgery; they may be suspected of having brain tumors. The primary aim of our study was to evaluate the clinical characteristics of pathologically confirmed demyelinating lesions that were easily confused with brain tumors (which we call atypical tumefactive demyelinating lesions) and to identify the risk factors of poor prognosis.

## Method

The present study was a retrospective review of patients presenting with TDLs as their first clinical event. We identified patients who underwent brain biopsy or neurosurgery at Tianjin Huanhu Hospital between January 2006 and December 2017 and received a final neuropathological diagnosis of TDLs. Patients who could not be followed up were excluded. Cases diagnosed with neoplasms, ADEM (acute disseminated encephalomyelitis), NMOSD, infection, vascular, and other fatal diseases were excluded.

The Tianjin Huanhu Hospital Ethics Committee on Human Research approved the study protocol for this retrospective review.

We recorded the following details: previous history, age, sex, race, CSF analysis results if it could be obtained, radiographic spectrum, clinical manifestations, treatment, and all the patients were followed up. Clinical information was obtained by board-certified neurologists, and the neuroimages were obtained by board-certified radiologists. The certified neuroradiologist who evaluated the radiographic material was blinded to the clinical data. We recorded the following neuroimaging details: lesion location, lesion number, degree of mass effect (mild, (sulcal effacement); moderate (ventricular compression); or severe (midline, sub-falcine, or uncal herniation)), degree of edema (mild: < 1 cm from the lesion; moderate:1–3 cm from the lesion; severe: > 3 cm from the lesion)^11^, and enhancement pattern (nodular, patchy, radiating, linear, and complete rim).

Stereotactic puncture biopsy and exploratory craniotomy were performed by a professional neurosurgeon. Informed consent have been obtained from all subjects and/or their legal guardians. The samples were obtained from the lesions with significant enhancement on MRI. All the cases diagnosed as TDLs during pathological examination were identified by 2 professional pathologists. All the cases underwent routine sections, special stains, and immunohistochemical studies.

The follow-up was conducted through personal interviews, examinations, telephone contacts, and family contacts. The EDSS were assessed at first onset and during follow-up in patients with TDLs.

### Ethical approval

All human studies have been approved by the appropriate ethics committee and have therefore been performed in accordance with the ethical standards laid down in the 1964 Declaration of Helsinki and its later amendments.

### Statistical analysis

We performed descriptive statistics as the statistical analysis.

## Result

As shown in Table 1. Eleven patients were diagnosed with TDLs by brain biopsy or neurosurgery. The ages of the patients ranged from 29 to 62 years, with a mean age of 48.9 years; 72.7% were males. The average EDSS of the patients at first onset was 2.36. Among these patients, there were five patients whose EDSS was 1, three patients whose ESSS was 3, three patients whose EDSS was 4, and all of the patients’ EDSS was ≤ 4. Regarding clinical symptoms (chief complaint on admission), there were five patients with headache or dizziness, seven patients with numbness or weakness of the limb, four patients with alalia, one patient with epilepsy, one patient with memory deterioration, and one patient with eye movement disorders. Five patients had a history of hypertension, two had a history of diabetes, one had a history of colonitis, one had a history of cholangiolithiasis, one had a history of duodenal ulcer, and one had a history of viral encephalitis. In conclusion, most of the patients with atypical TDLs were middle-aged (mean age 48.9 years), did not have any serious nervous system injury (EDSS ≤ 4), most started with limb numbness and weakness (45.5%) or alalia (27.2%). The mean time from symptom onset to biopsy or surgery was 12.9 days  (3–30 days).

**Table 1:** Basic information of 12 pathologically diagnosed TDLs patients.

Patient	Sex	Time of onset	Age	Clinical symptoms	EDSS of onset	Anamnesis	Lesions	Edema	Mass effect
1	Female	2014	61	Headache; weakness of limb	3	Hypertension diabetes	Multifocal (Right frontal and parietal lobe)	Mild	Mild
1 (relapse)	Female	2018	65	Alalia; weakness of limb	4	Hypertension; diabetes	Solitary (pons)	Mild	Mild
2	Female	2006	56	Headache;numbness of limb	3	Health	Multifocal (Left frontal, temporal,right temporal)	Moderate	Moderate
3	Male	2012	57	Dizziness, memory deterioration	1	Colonitis	Solitary (Right frontal, corpus callosum, basal ganglia)	Moderate	Moderate
3 (relapse as MS)	Male	2013	57	Weakness of limb; Eye movement disorder	7.5	Colonitis	Solitary (brachium pontis)	Mild	Mild
4	Male	2015	48	Alalia	1	Cholangiolithiasis	Solitary(left temporal parietal lobe)	Moderate	Moderate
5	Male	2018	37	Alalia	1	Health	Solitary (left temporal )	Moderate	Moderate
6	Male	2012	55	weakness of limb	3	Hypertension	Solitary (left frontal lobe)	Moderate	Mild
7	Male	2013	62	Headache and dizziness	1	Hypertension	Solitary (Right basal ganglia)	Moderate	Mild
8	Male	2012	49	weakness of limb	4	Hypertension, duodenal ulcer	Solitary (Left of centrum semiovale)	Mild	Mild
9	Male	2009	50	Numbness and weakness of limb	4	health	Solitary (left centrum semiovale)	Moderate	Mild
10	Female	2008	34	Alalia	4	viral encephalitis	Multifocal (pons, midbrain and left thalamus)	Mild	Mild
11	Male	2013	29	Epilepsy	1	Healthy	Solitary (left frontal,parietal)	Sever	Sever

Patient	Enhancement	Function -MRI	MBP Or MOG	The time from onset to biopsy or surgery	Preoperative diagnosis	Frozen pathology	Treatment	Prognosis	EDSS of follow-up
1	Patchy and nodular	–	MBP(−)	4 days	Brain tumor (metastatic tumor)	Most foam cells are infiltrated with local glial hyperplasia	Exploratory craniotomy, Glucocorticoids,	Relapse(4 years later)	4 (4 years later)
1 (relapse)	None	–	–	–	–		Immune globulin therapy	No recurrence again	4 (3 years later)
2	None	–	–	14 days	Brain tumor (metastatic tumor, or TDL)	Edema brain tissue, less inflammatory cells	Exploratory craniotomy, Glucocorticoids,	No Recurrence	1 (14 years later)
3	Patchy	–	–	30 days	Brain Tumor(glioma or TDL)	Edema brain tissue, less inflammatory cells	Exploratory craniotomy, Glucocorticoids,	Recurrence (MS,2 Monthes later)	1
3 (relapse as MS)	None	–	MBP (+)	–	MS	–	Immune globulin therapy, Glucocorticoids	During the treatment, limb weakness was aggravated and epileptic seizure was happened	10 Died in 2015 (2yearslater)
4	Complete rim	MRS: NAA decrease significantly, Cho Slightly reduced, Cr decrease significantly, Cho/NAA increase)	–	30 days	Tumor (TDL or tumor)	TDL	Exploratory craniotomy, Glucocorticoids, Immunosuppressive therapy	No recurrence again	1 (5 years later)
5	Patchy	Mrv (−)	–	7 days	Low-grade gliomas	Glial cell proliferation and more foam—like cells	Exploratory craniotomy	No recurrence again	1 (2 years later )
6	None	–	–	15 days	Low-grade gliomas or TDL	Glial hyperplasia with demyelination changes	exploratory craniotomy	No recurrence again	3 (8 years later)
7	Patchy	–	–	7 days	Gliomas	Mass foam cell	Exploratory craniotomy	No recurrence again	0 (7 years later)
8	Complete rim	–	MBP (+)	3 days	TDL	Foam cells and inflammatory cell infiltration	Brain biopsy. Glucocorticoid	10 days Later, lesions increase	1 (8 years later)
9	Patchy	–	–	15 days	Brain tumor	Heterotypic cells are seen	Exploratory craniotomy	Seizures, limb weakness aggravated after operation	3 (11 years later)
10	Patchy	–	MBP (+) MOG (+)	7 days	Tumor or TDL	–	Exploratory craniotomy Glucocorticoid	No recurrence	0 (12 years later)
11	Linear	–	MBP (−)	10 days	Tumor (glioma)	Star cell tumor grade 2–3	Exploratory craniotomy, Glucocorticoid	Postoperative brain abscess	10 Died in 2015 (2 years later)

**Figure 1:**
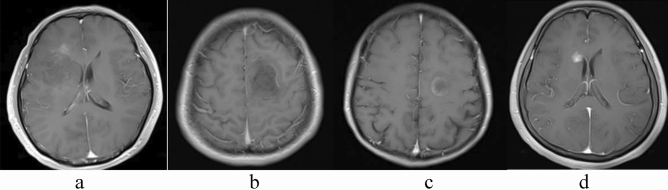
Representative examples of different enhancement patterns: (**a**) patchy (**b**) linear (**c**) Complete rim (**d**) nodular.

In terms of imaging, there were eight patients with solitary lesions (72.7%) and three patients with multifocal lesions (27.3%). Only one (9.1%) of the 11 patients had sub-tentorial lesions, three patients (27.2%) had right hemispheric lesions, six patients (54.5%) had left hemispheric lesions, and 1 patients (9.1%) had bilateral hemispheric lesions. Frontal lobes (5 cases, 45.5%), temporal lobes (3 cases, 27.3%), and parietal lobes (3 cases, 27.3%) were the most prevalent lesions. Mild edema surrounded the lesions in three cases (27.3%), moderate edema surrounded the lesions in 7 patients (63.6%). Six patients had mild mass effects (54.5%), four patients (36.4%) had a moderate mass effect. Enhancement following the administration of gadolinium was observed. There were six patients whose lesions were patchy (Fig. 1a) in MRI enhancement (54.5%), no patients with incomplete rim lesions, one patients with linear lesions (Fig. 1b), two patients with complete rim lesions(Fig. 1c), one patient with nodular lesions (Fig. 1d) and two patient with no enhancement of the lesion. No patients had spinal lesions. In conclusion, most of the patients had solitary lesions (72.7%), supratentorial lesions (90.9%), in particular, the frontal, temporal, and parietal lobes were predominant), moderate edema (63.6%), mild mass effect (54.5%), and patchy lesions (54.5%).

Most of the patients were considered to have intracranial tumors before surgery; therefore, few cerebrospinal fluid analyses and related demyelination tests were performed before surgery. Only four patients underwent lumbar puncture and related demyelination test. Among them, three patients were positive for MBP, one patient was negative for MBP, and one patient was positive for MOG.

Of the 10 patients who underwent frozen pathology, two patients were considered as having brain tumor in frozen pathology, seven patients could not be determined as having TDLs or tumors, and only one patient was considered as having TDLs in frozen pathology. Five cases were considered as tumors before surgery, five cases could not be determined as having tumors or TDLs and the symptoms and imaging findings did not improve after immunotherapy, one case whose cerebrospinal fluid was positive for MOG that was considered as having TDLs, but the symptoms worsened after hormone therapy; therefore, surgery was performed.

Regarding the treatment, five patients underwent exploratory craniotomy and hormone therapy; one patient underwent exploratory craniotomy, hormone therapy, and immune globulin therapy; 4 patients underwent exploratory craniotomy with complete removal of the lesions in the following MRI examination; therefore, these 4 patients were not given hormone or immunoglobulin therapy. One patient underwent brain biopsy and hormone therapy. The patients were followed up for an average of 6.9 years (2–14 years), two patients relapsed, and one of them died after 2 years. Of the other 9 patients, one died after 2 years, and the other 8 patients recovered very well (the EDSS scores decreased or did not change) and did not develop new lesions over time. The average EDSS of the 8 patients was 1.25 (range, 0–3). Among the 8 patients, 4 patients underwent only neurosurgery, two underwent exploratory craniotomy and hormone therapy, one patient underwent exploratory craniotomy, hormone therapy, and immunoglobulin globulin therapy, and one patient underwent brain biopsy and hormone therapy.

**Figure 2:**
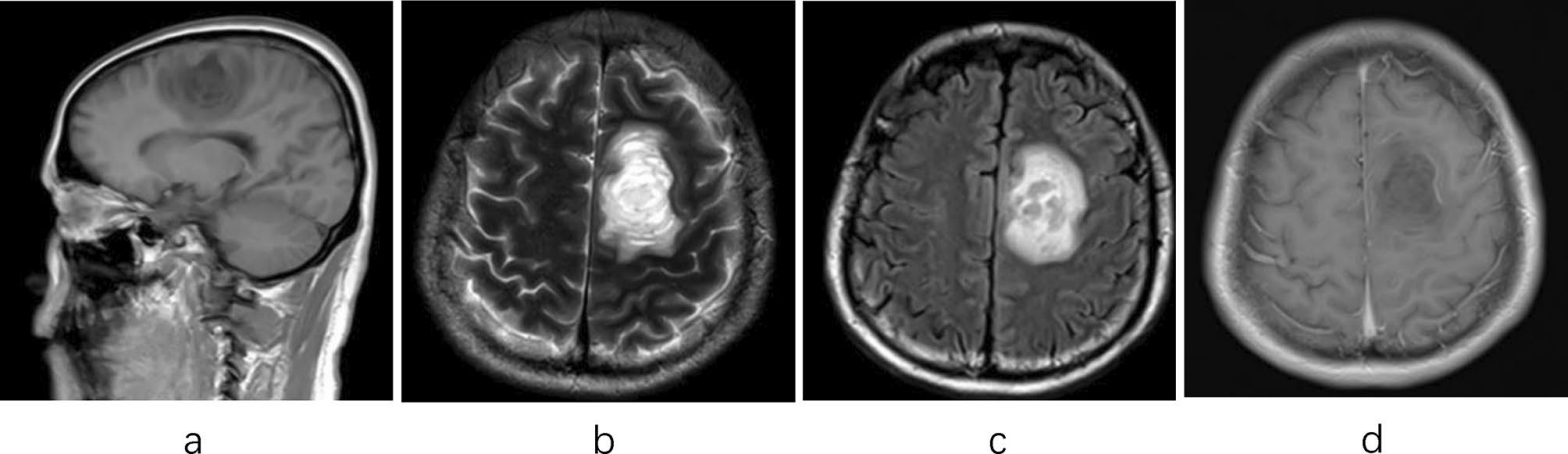
Male, 29 years old, 10 days of paroxysmal convulsion and 10 days of dizziness. (**a**) T1 sagittal position: left frontoparietal long T1 signal; (**b**) T2 axial position: left frontoparietal long T2 signal; c Flair axial position: left frontoparietal high signal ;d enhanced NMR axial position: linear enhancement.

There were two epileptic patients, one of who was a 29-year-old male with 10 days of paroxysmal convulsion and 10 days of dizziness. Head MRI showed a space-occupying lesion on the left frontal parietal (Fig. 2) with irregular morphology and linear enhancement. Pathological findings showed that a large number of foam cells were deposited in the local brain tissue of the test material, with myelin loss, axonal retention, peripheral glial cell proliferation with small vascular hyperplasia, lumen dilation, and focal vascular lymphatic sleeve formation, which was considered as demyelinating pseudotumor.

**Figure 3:**
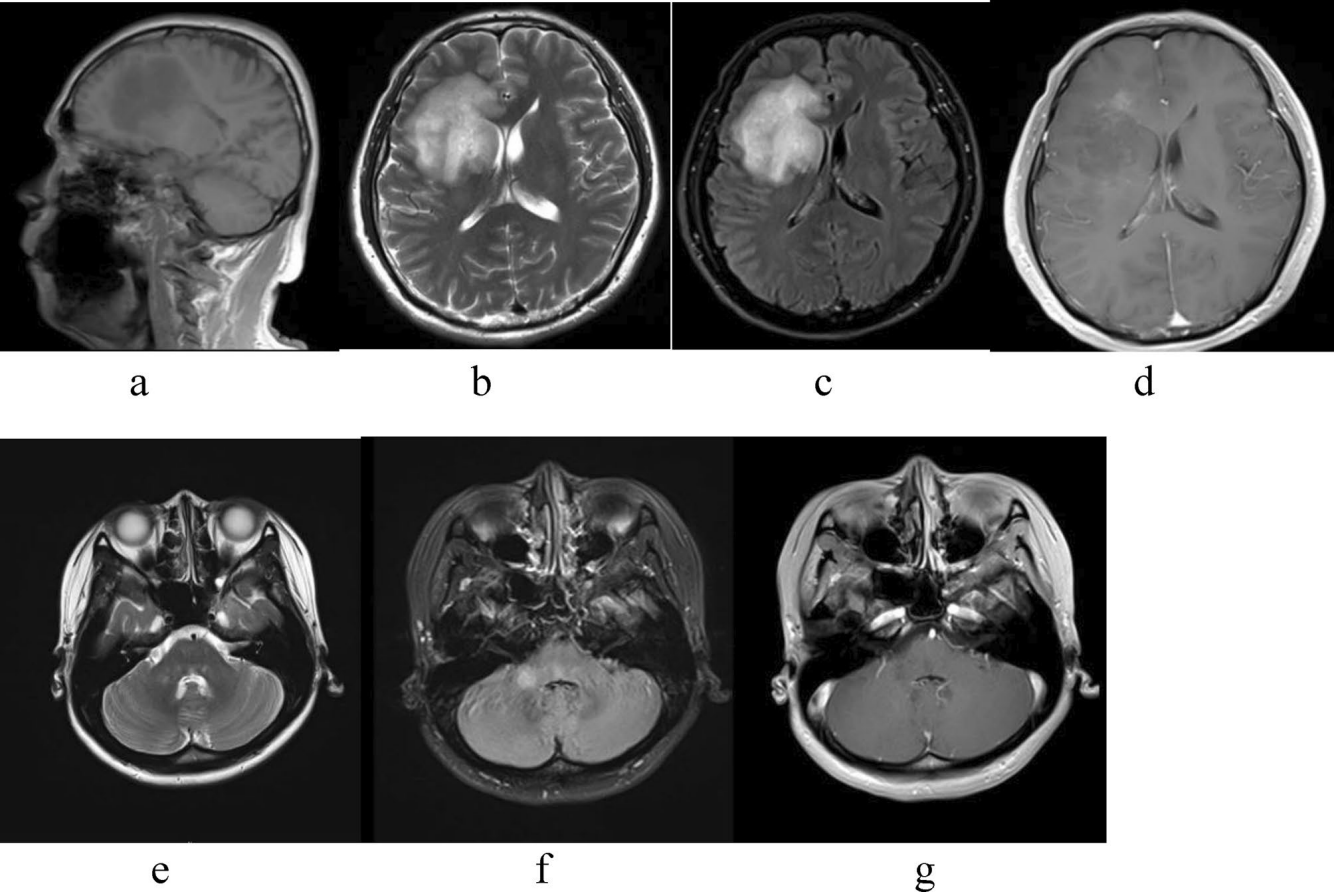
Male, 57 years old, dizziness and memory loss for more than 1 month (**a**–**d**). The patient relapse after 1 year, weakness of left limbs and seizures (**e**–**g**). (**a**) T1 sagittal position: right frontal lobe, corpus callosum, basal ganglia, and periventricular long T1 signal; (**b**) T2 axial position: right frontal lobe, corpus callosum, basal ganglia, and periventricular long T2 signal; (**c**) Flair axial position: right frontal lobe, corpus callosum, basal ganglia, and periventricular high signal; (**d**) enhanced MRI axial position: patchy enhancement. The patient relapse after 1 year (**e**–**g**). (**e**) T2 axial position: right brachium pontis long T2 signal; (**f**) Flair axial position: right brachium pontis high signal; (**g**) enhanced MRI axial position: none enhancement.

The other patient was a 57-year-old male patient with dizziness and memory loss for more than 1 month. MRI of the head (Fig. 3) showed that the right frontal lobe, corpus callosum, basal ganglia, and periventricular lesions with irregular morphology, unclear boundary, and insignificant peripheral edema. MRI enhancement of the head shows patchy enhancement. Pathological examination showed a large number of foam cell deposits in brain tissue, scattered lymphocytes and plasma cells in infiltration and perivascular cuff cluster, accompanied by a large number of astrocyte proliferation, which was considered demyelinating disease. The symptoms improved after hormone therapy. One year later, the patient developed increased limb weakness and recurrent seizures, and the MRI showed abnormal signals in brachium pontis (Fig. 3). He was diagnosed as MS. After immunoglobulin and hormone therapy, symptoms worsened. The patient's family gave up treatment and died two years later.

Recurrent TDLs were observed in two patients. One patient relapsed 3 years after the exploratory craniotomy and hormone therapy. The recurrent lesion in this patient was located in the pons, different from the first lesion. After immune globulin therapy, the patient recovered very well within 3 years of follow-up. The other patient relapsed 2 months after the exploratory craniotomy and hormone therapy. The recurrent lesion in this patient was located in brachium pontis, different from the first lesion. Immune globulin therapy was administered. The patient died after 2 years. The lesions of the patients who relapsed were solitary. There were no enhancement in both two recurrent patients. Except for the two patients who relapsed, only one patient of the 9 patients died, and the other 8 patients improved or maintained the status quo (the EDSS scores were lower or did not change). Three patients maintained the status quo and the other five patients improved. Among the 8 patients, four patients underwent exploratory craniotomy and other immunotherapies, while 4 patients underwent only exploratory craniotomy.

**Figure 4:**
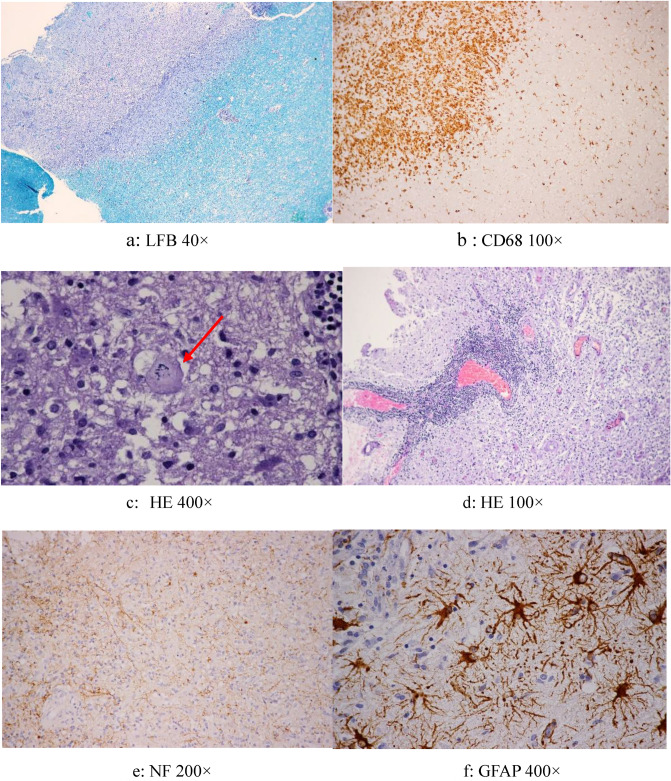
Pathologic features (**a**) Discrete border and demyelination (**b**) Discrete border and Macrophage infiltrate (**c**) Creutzfeldt cells (red arrows) (**d**) Perivascular lymphocytic inflammation (**e**) Preserved axons (**f**) Reactive astrocytes.

Postoperative paraffin pathological findings showed discrete border, different degrees of demyelination in the lesion area (Luxol-fast-blue, LFB) (Fig. 4a) ,macrophage infiltration (CD68) (Fig. 4b), Creutzfeldt cells(Fig. 4c), perivascular lymphocytic inflammation(Fig. 4d), preserved axons (neurofilament protein immunohistochemistry, NF) (Fig. 4e) and reactive astrocytes (glial fibrillary acidic protein, GFAP) (Fig. 4f). A final diagnosis of TDLs was made in all the cases. The myelin loss in the areas of macrophage infiltration and the preservation of axons in the areas of demyelination are supportive histological findings in TDLs.

## Discussion

TDLs were first described in 1979 by van der Velden et al.^12^ It has characteristic clinical and radiographic features, sizes ˃2 cm , incomplete annular enhancement, small edema and mass effects, and experienced neurologists can diagnose typical TDLs in most cases. However, there are no consensus criteria for the clear definition of TDLs, and the 2-cm cut-off for the definition of TDLs is an arbitrary one. One report found that when the TDLs were grouped into < 2 cm and > 2 cm, there was no difference in clinical features and MRI manifestations^13^. In our study, we included 11 patients, of which 5 patients (45.5%) were considered to have tumors before the surgery, and for 5 patients (45.5%), it was not sure whether they were tumors or TDLs and had poor response to immunotherapy, and one (9.1%) case was considered as TDLs, but the symptoms worsened after hormone therapy. In all the cases, it was difficult to establish the diagnosis of TDLs or tumors; therefore, a biopsy or surgery was advisable. Consequently, these cases were histopathologically proven to be a demyelinating disease. This suggests that it is difficult to completely differentiate TDL from a tumor, and pathological biopsy is still the gold standard for the diagnosis of TDLs. Misdiagnosis can result in unwarranted and overly aggressive therapy; therefore, it is critical to be aware of this serious diagnostic pitfall. TDLs have no biomarker or indicator, and the prognostic value of TDLs is uncertain. Therefore, we summarized the clinical and radiological features and prognostic factors of atypical TDLs and hoped to find clues as to why some patients developed TDLs.

TDLs is estimated to have an annual incidence of 0.3/100,000^14^. The figure is likely an underestimation because of under-reporting**,** and the true incidence is unknown. TDLs can occur at any age (2–71 years)^8,15,16^, but persons in their 20 s and 30 s were more frequently affected^1,17^. In our study, the ages of the patients ranged from 29 to 62 years, with a mean age of 48.9 years. The mean age was higher than for previous studies due to the number of atypical cases and the small number of cases. Female predominance has been reported in previous reports^17–19^. However, research including 168 cases of pathologically confirmed tumefactive MS observed no gender predilection, which is consistent with our results^1^.

The clinical manifestations of TDLs vary depending on the location and size. TDLs generally have an acute onset and gradually aggravate. In our study, the patients did not have serious nervous system injury (EDSS ≤ 4) at onset; the duration from onset of symptoms to biopsy or surgery was mostly within 30 days, and the main presentation included weakness of the limbs, headache or dizziness, and alalia. Cognitive impairments and seizures have also been reported^1,20^; one patient presented with a seizure and one patient with cognitive impairments in our study. There was also one patient with eye movement disorders. These findings suggest that the disease is insidious and that these clinical manifestations are indistinguishable from tumors. Our research did not involve children, but some studies found that TDLs have a relatively acute onset (mostly less than 1 weeks) in children, and symptoms are severe, including headache and vomiting because of intracranial hypertension^15,16^. The most common lesions of TDLs are located in the subcortical hemispheric white matter and corpus callosum^8,21–23^. TDLs lack a mass effect and less perifocal edema^21^, but the mass and perifocal edema increases with larger lesions and in a more acute onsets(< 3 weeks) ^1,19,24^.TDLs are generally thought to be solitary lesions^8,21^. There are various patterns such as nodular‑,closed‑ring‑, open‑ring‑, and flame‑shaped enhancements that are noted in strengthen MRI^25^.The patterns of incomplete, C-shaped, or “open-ring” enhancements are considered to be typical TDLs imaging findings. In particular, the open-ring sign was present in 70% of the demyelinating disease^26^. The open portions of the open-ring face the cortical gray matter or basal ganglia and the portions of enhancement face the white matter^27^.The lesions of enhancement are regarded as demyelination and the lesions that are not enhanced are regarded as chronic inflammation^28^. In our study, all the cases appeared as hypointensities on T1-weighted MRI and hyperintensities on T2-weighted MRI. The lesions in most of the patients were solitary, supratentorial, and unilateral (there was no obvious distinction between left and right hemispheric) lesions, and the frontal, temporal, and parietal lobes were the most prevalent locations of the lesions, which is consistent with the results of some studies^1,19^.

Most of the patients in our study had moderate edema and mild mass effect; that is, neither the edema nor the mass effect was serious. The characteristics mentioned above are consistent with the literature reports. However, the forms of MRI enhancement in atypical TDLs were inconsistent with the literature, and the most common form was patchy enhancement, which was confused with tumors. There was no incomplete rim in the MRI enhancement. The reason would be that the typical TDLs with incomplete rims were diagnosed and had avoided surgery. The atypical TDLs without incomplete rims are easily confused with tumors. TDLs could also occur in the spinal cord, and cervical lesions appear to be the most commonly occurring spinal lesion^29–32^. The cases in our study did not include spinal lesions.

Sometimes, conventional MRI imaging was unable to discriminate between demyelination and neoplastic processes. Special imaging examinations such as magnetic resonance spectroscopy (MRS), MRI perfusion, and positron emission tomography (PET) are helpful for the identification of TDLs. In MRS, TDLs have decreased N-acetylaspartate peaks/Cr ratios and increased Cho/Cr^33^. A study found that dilated veins drain towards distended subependymal veins in MRI perfusion studies^34^. The mean regional cerebral blood volume (rCBV) in TDLs was lower than the mean rCBV in the contralateral normal-appearing white matter and substantially less than in gliomas^34,35^. In PET-CT, TDLs have lower metabolic activity than neoplasms^36^. However, advanced MRI techniques can lead to equivocal results because there is a broad overlap between the TDL and tumors. Unfortunately, only two of our patients underwent functional MRI. The magnetic resonance venogram (MRV) of one patient was normal and the MRS of the other suggested a high possibility of TDLs, but a glioma could not be excluded. As the sample size was small, we could not summarize the role of the special imaging examination. Further research is required to clarify this is needed.

Typical TDL are open loop enhancement and vertical sign (linear enhancement perpendicular to the lateral ventricle). Our atypical TDL are all non-open loop enhancement, and there is no vertical sign. Most of our atypical TDL enhancement were patchy and nodular reinforcement, and some of them had no significant enhancement changes. If the imaging findings are confused with tumors, we recommend further examination, for example, magnetic resonance perfusion examination. Although the enhancement of TDLs is obvious, it often presents equal or low perfusion due to the absence of tumor neovascularization, while neoplastic lesions, such as glioblastoma, often present obvious high perfusion. MRS Examination is also helpful in the diagnosis of TDLs.

The TDLs were dynamic, consistent with the disease progression^37^. Most TDLs lesions showed an excellent response to hormone therapy, especially on diffusion-weighted imaging (DWI), and there was a substantial decrease in size or disappearance of the lesions after 6–8 weeks, which is in contrast to the more stable findings in patients with tumors^8,38,39^.Therefore, the dynamic changes of the image is a feature that differentiates TDLs from tumors.

The patients with TDLs had slightly elevated concentrations of cerebrospinal fluid (CSF) protein or mild CSF pleocytosis^40^. The myelin basic protein (MBP) may play a role in TDLs^41,42^. One study found that oligoclonal band positivity was less frequent in patients with TDLs onset^19^. The importance of anti-MOG testing in NMO spectrum disorders and other demyelinating disease phenotypes, including TDLs, has since been highlighted in emerging research^43^. Some reports showed that TDLs with MOG antibody positivity had MS-like histopathological features and had a good response to immunotherapy^44^. Some studies found that patients with NMOSD who are AQP4-IgG-seropositive or myelin oligodendrocyte glycoprotein (MOG)-IgG-seropositive can develop TDLs^45–47^. One study found that the conversion of TDL to aquaporin-4 AQP4-NMOSD is increased^47^. In our study, 3 patients had MBP positivity and one patient had both MBP and MOG antibody positivity. One patient with MBP positivity evolved into MS 4 years later. The patient with both MBP and MOG antibody positivity did not develop MS or NMOSD. In our study, although the sample size was small, the cerebrospinal fluid and demyelination tests could be an indicator of TDL. The absence of oligoclonal bands or other markers of intrathecal inflammation cannot exclude the demyelination disease^48^. More studies on the demyelination test will be needed.

The histologic features, particularly those observed on intraoperative frozen sections, can be deceptive and difficult to interpret, and they often lead to the misdiagnoses of TDLs as astrocytomas because it is difficult to distinguish macrophages from swollen astrocytes. As seen in this study, only one of the frozen sections suggested a diagnosis of TDLs. However, the CD68 marker can distinguish between macrophages and swollen astrocytes. In our study, for most of the patients (7/10, 70%) in the intraoperative frozen section, it could not be determined whether they were TDLs or tumors, and some of them were diagnosed as tumors (2/10, 20%) in the intraoperative frozen section; only one of them (1/10, 10%) was diagnosed as TDLs in the intraoperative frozen section, while postoperative paraffin section confirmed the TDLs. The accuracy of frozen pathology in diagnosing TDLs is relatively low. Therefore, in the process of craniotomy exploration, our patients underwent total resection without affecting the neurological function as far as possible. Brain biopsy may sometimes lead to misdiagnosis. Due to sampling bias of the tissue biopsied, misdiagnosis can occur after pathological confirmation. Biopsy samples may be insufficient or nonrepresentative of tissue compounds. The size, quality, and site of the biopsy, fixation conditions, and other factors can affect the accuracy of the pathological results. Experienced interpretations of neuropathologists and neurologists are critical. In our study, the patient who was diagnosed with TDL only before surgery underwent brain biopsy, while the others underwent exploratory craniotomy. During the operation, all the lesions were removed as far as possible.

The treatment of TDL is based on the findings from case reports and series, and there are no recognized recommendations. TDLs respond well to steroid hormones^49^, corticosteroids are the usual first-line treatment, as in conventional MS^19^. If patients on hormonotherapy recover incompletely or worsen, plasma exchange (PLEX) is an appropriate next-line therapy^50^. If the symptoms of TDLs worsen and the lesions enlarged after hormonotherapy and PLEX, immunosuppressive therapy should be started without hesitation^17^. If brainstem herniation seems to be imminent due to increased intracranial pressure, decompressive craniectomy should be strongly considered^51^. If the patients with TDLs are subsequently diagnosed with MS or NOSDM, the standard immunotherapies for those conditions should be considered. We had four treatments: exploratory craniotomy only, exploratory craniotomy combined with hormone therapy, exploratory craniotomy combined with hormone therapy and immunoglobulin therapy, brain biopsy, and hormone therapy. No patient received PLEX. The 4 patients who underwent exploratory craniotomy recovered very well (the EDSS scores decreased or did not change) and did not develop new lesions over time. The patient who underwent exploratory craniotomy combined with hormone therapy and immunoglobulin therapy as well as the patient who underwent brain biopsy and hormone therapy recovered well and did not develop new lesions over time. However, among the 5 patients who underwent exploratory craniotomy combined with hormone therapy, 2 patients relapsed and one of them died after 2 years. The lesions of the patients who experienced relapse were both located in the pons. One patient was diagnosed with TDLs after the relapse and had a good prognosis. Another patient was diagnosed with MS and died 2 years later. The patient died due to a worsening of the disease with seizures and complications of lung infection. One patient died 2 years after the exploratory craniotomy and hormone therapy. Due to the large lesion, frequent epileptic seizures, and economic reasons, the gamma globulin treatment was not applied. The 2 patients who died both had epileptic seizures; therefore, the seizures may be a poor prognostic indicator.

The lesions in two patients with epilepsy were close to the cortex, had space-occupying effects and edema, and were prone to epilepsy. Pathological findings showed foam cell deposition, disappearance of myelin sheath, retention of axons, scattered infiltration of lymphocytes and plasma cells and perivascular cuff aggregation, accompanied by massive proliferation of astrocytes, all of which were considered demyelinating disease.

One of the two patients complained of epilepsy, while the other was admitted with dizziness and memory loss and later recurrent limb weakness and seizures, which were not isolated seizures. Epilepsy is rare in TDLs. In a case series study, epilepsy was associated with hemiplegia in 2 out of 18 patients with TDLs^52^.In another study, epilepsy occurred in 6% of patients with TDLs^1^. An Indian child was also reported to have suffered epilepsy as well as hemiblindness and mild hemiplegia^53^. There has also been a report of patient with TDLs presenting with epilepsy as an isolated manifestation^54^. Therefore, epilepsy is rare in TDLs patients, most of which are combined with other signs of neurological impairment. However, TDLs characterized by isolated seizures epilepsy are relatively rare.

The recurrence rate of TDLs has been extremely variable (10–70%) ^1,7,10,19,48,55^.The risk of relapse or imaging progression between patients with solitary TDL and patients with multiple lesions is similar^48^. The recurrence rate of TDLs in our group was 16.7% (2/12). In our group, one patient recurred as MS; therefore, the TDLs were associated with MS or NOMDS. Some TDLs have a preexisting diagnosis of MS^17,56–60^. Some studies have suggested that TDLs may represent either a unique form of demyelinating disease^8^ or a variant of MS^61^. TDLs are sometimes regarded as Shilder's disease or Marburg's variants^61^. TDLs can occur as part of other demyelinating diseases: NMOSD or acute disseminated encephalomyelitis (ADEM)^1,62^. One report found that most patients followed the typical course of MS, while a small subset developed relapsing TDLs^1^. Therefore, it may also be necessary to further determine the relationship between TDLs, MS, or NMOSD.

There are no definitive diagnostic criteria for TDLs. Some of the TDLs have no pathognomonic imaging or clinical manifestations. Currently, biopsy cannot be replaced by imaging in the diagnosis of TDLs. If the radiology is uncertain and the clinical features are atypical, we can attempt to use steroids or plasma exchange for treatment. A response to the treatment is particularly suggestive of an inflammatory demyelinating lesion. We suggest to avoid unnecessary surgical treatment and to choose drug control first. However, when surgery is unavoidable, total surgical resection without affecting the neurological function is recommended. Postoperative immunotherapy will be used according to the situation. In patients with mild or improved symptoms, it can be considered not to use immunotherapy because the lesion has been completely excised during the surgery. But this idea is still controversial. Although The effect of neurosurgery alone was good in our group. But in our study, half of the patients who underwent surgery had tumors considered preoperatively, and half were patients who did not respond well to immunotherapy (hormone or hormone combined with propyl bulb). So, this may have something to do with our selection bias. In our group, the effect of surgical excision alone was still good. Among the 5 patients who underwent exploratory craniotomy combined with hormone therapy, 2 died with a poor prognosis, which may be related to the aggravation of the disease, and it is not necessarily related to the use of hormones. The samples size is small; therefore, large sample studies are still needed to confirm this. The effect of neurosurgery alone was good; therefore, the effect of surgery on TDLs can be further studied.

Most TDLs may have a possible benign course, and they are not associated with poorer outcomes^1,8,63^. Some reports suggested that patients with TDLs often had a monophasic and benign course^16,64^. The prognosis of patients with TDLs is relatively more favorable than expected in Taiwan. Most of the patients were females (11/12), and all the patients had relapsing-remitting^10^ .One report found that patients presenting with isolated TDLs might have a better long-term prognosis than patients with conventional MS^6,65^, which was consistent with our results. Patients who experienced recurrence as MS died in our study. Most of the other patients had a good outcome.

Limitations of our study include the relatively small sample size, incomplete inspection, and some missing data, given that this was a retrospective review. Children with TDLs were not included in our study. Our study did not include spinal lesions. Most of the patients were unable to undergo an imaging examination. The strength of our study is that we included pathologically proven cases.

In the future, we need further large-scale studies comparing suspected TDLs cases that did not undergo pathological examination with the patients who underwent surgery, in order to conclude whether the surgery caused damage or was helpful for the treatment of patients. It may also be necessary to further determine the relationship between TDLs, MS, or NMOSD. This article serves as a starting point.

## Conclusion

There was no gender predilection of the atypical TDLs in our group. The patients did not have serious nervous system injury at onset, and the main presentation included weakness of the limbs, headache or dizziness, and alalia. Most of the lesions were supratentorial lesions, and the lesions were most prevalent in the frontal, temporal, and parietal lobes. Neither edema nor mass effect was serious in the cases in this study. The most common form was patchy lesions on MRI enhancement in atypical TDLs. Special imaging examinations are helpful for the identification of TDLs. Cerebrospinal fluid and demyelination test can be an indicator of TDLs, and seizure may be a poor prognostic indicator. The intraoperative frozen section can be deceptive. Most TDLs have monophasic courses and good outcomes. The effect of neurosurgery alone was good in our group, and the effect of surgery on TDLs can be further studied. Is total excision without affecting the neurological function a better option when surgery is unavoidable? This needs to be verified by a large number of further studies. It is necessary to follow-up the patients diagnosed with TDLs by brain biopsy and neurosurgery.

## Supplementary Information

Supplementary Information.

## Data Availability

The data that support the findings of this study are available from the corresponding author [YW], upon reasonable request.
