# Supplementary material for: Clinical spectrum and prognosis of pathologically confirmed atypical tumefactive demyelinating lesions
Source: Sci Rep. 2023 May 13;13:7773. doi: 10.1038/s41598-023-34420-4 (PMC10183015; doi:10.1038/s41598-023-34420-4)
Supplement: Supplementary file 1 — Supplementary Information. [file 41598_2023_34420_MOESM1_ESM.docx]

Supplementary material:


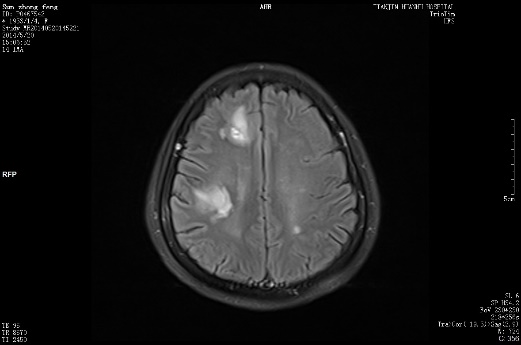

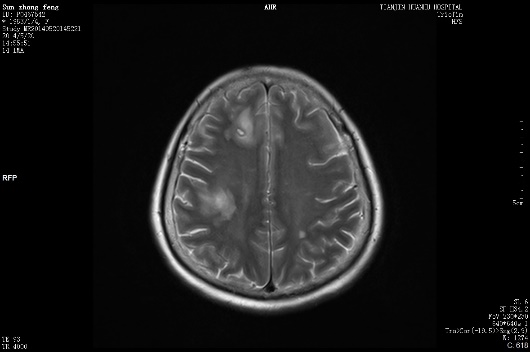

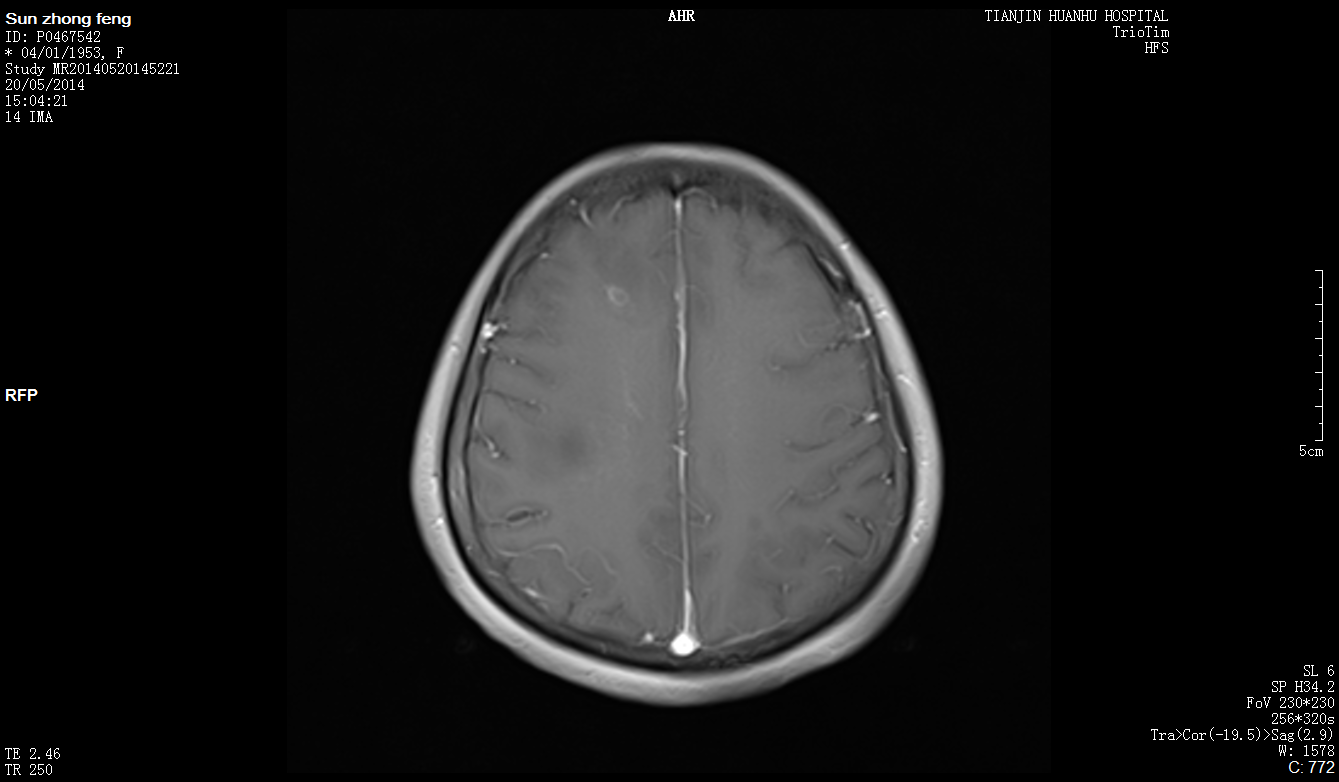


a b c


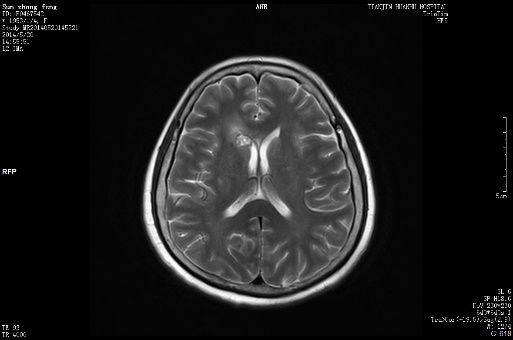

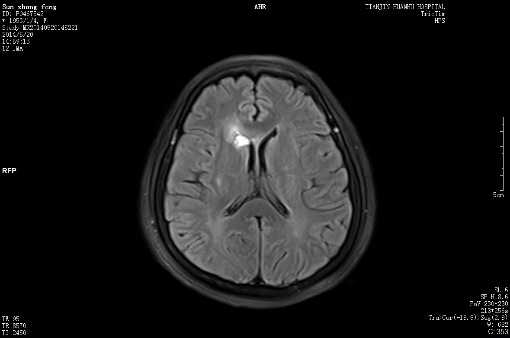

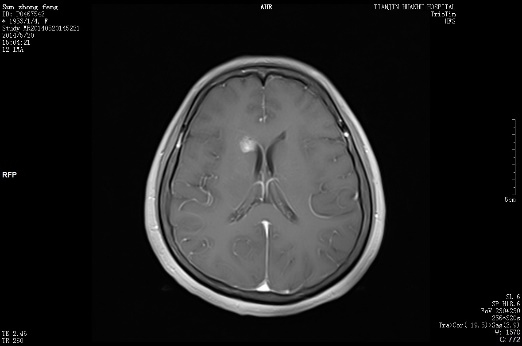


d e f

Figure S1: case 1, female, 61 years old, weakness of left limb and headache for 2 days. MRI: abnormal signals in the right frontal and parietal lobe.


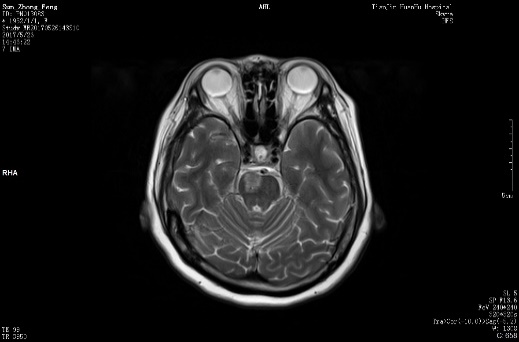

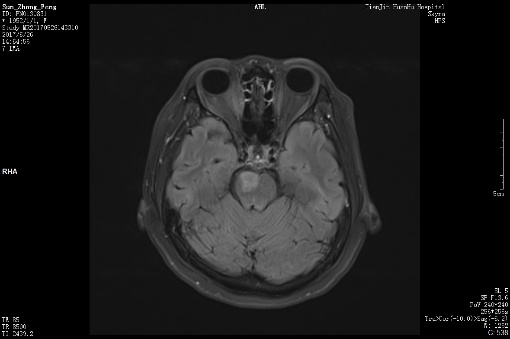

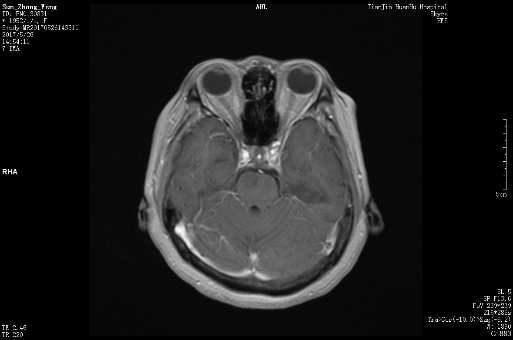


a b c

Figure S1(relapse): case 1(relapse), female, case 1 recurred 4 years later, alalia and weakness of left limb for 1 week**,** MRI: abnormal signals in the right pons**.**


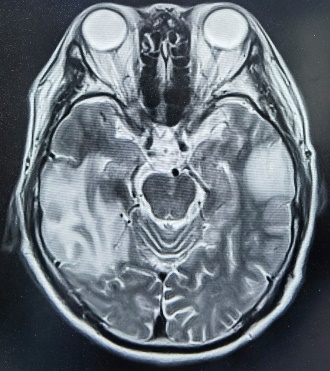

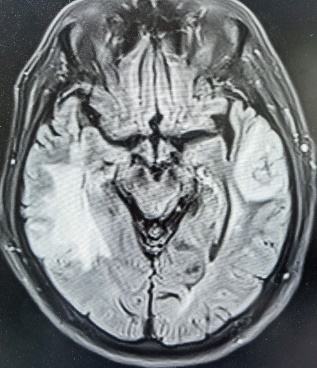

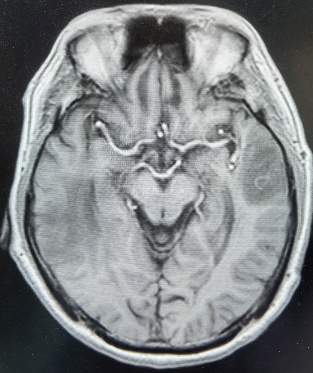


a b c

Figure S2: case 2, female, 56 years old, headache and numbness of right limbs for 2 weeks. MRI: abnormal signals in left frontal, temporal and right temporal.


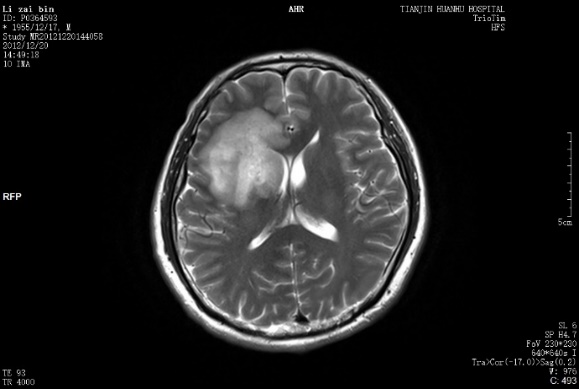

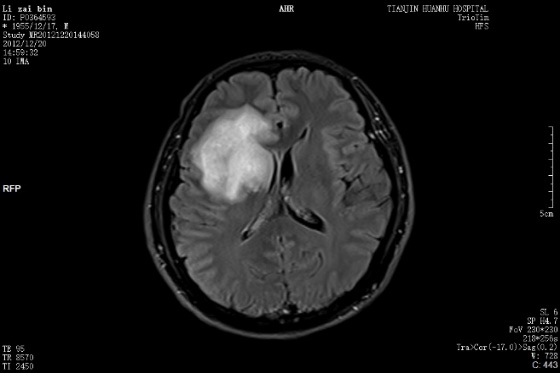

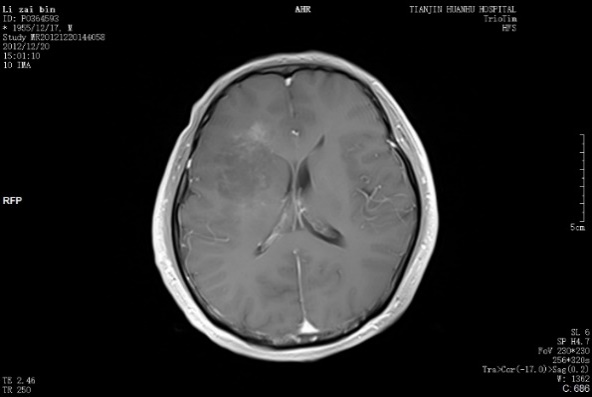


a b c

Figure S3: case 3, male, 57 years old, dizziness and memory deterioration for more than 1 month. MRI: abnormal signals in the right frontal lobe, corpus callosum and basal ganglia.


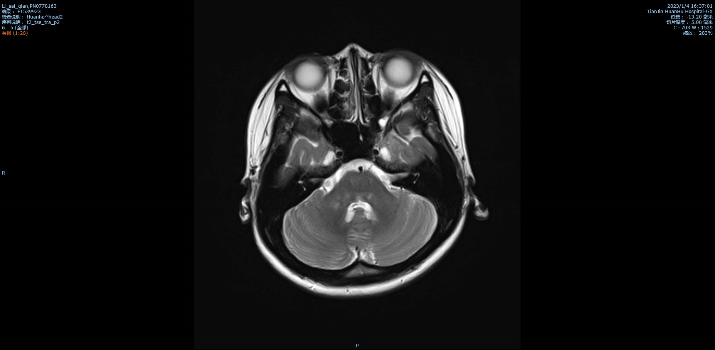

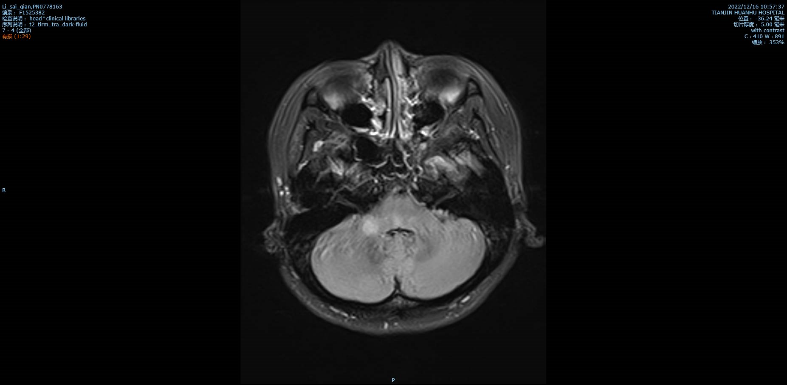

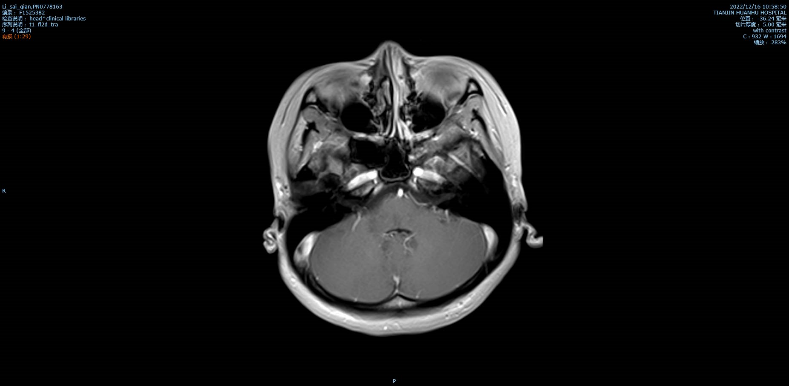


a b c

Figure S3(relapse): case 3(relapse), male, patient 3 relapse after 1 year, weakness of left limbs and eye movement disorder for 3 days. MRI: abnormal signals in [brachium pontis](javascript:;). She was diagnosed as MS. After immunoglobulin and hormone therapy, symptoms worsened and seizures occurred. The patient's family gave up treatment and died two years later.


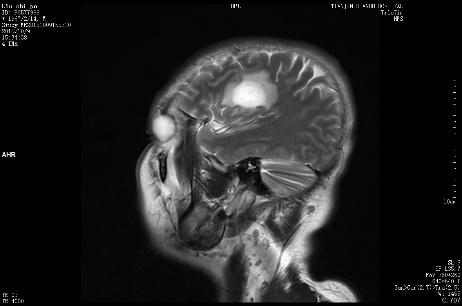

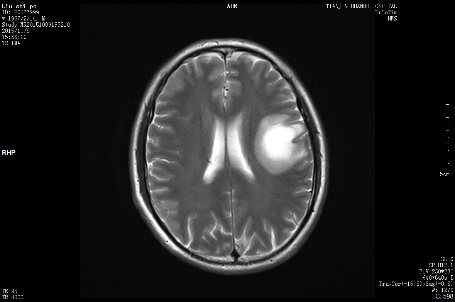

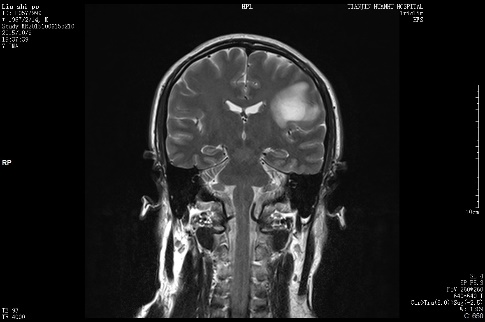


a b c

Figure S4: case 4, male ,48 years old, alalia for 2 weeks. MRI: abnormal signals in the left temporal and parietal lobe.


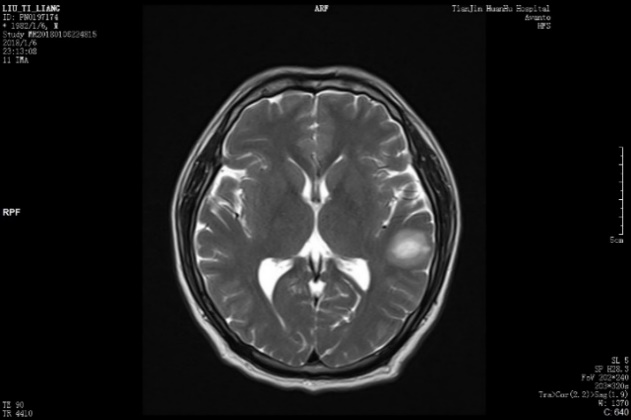

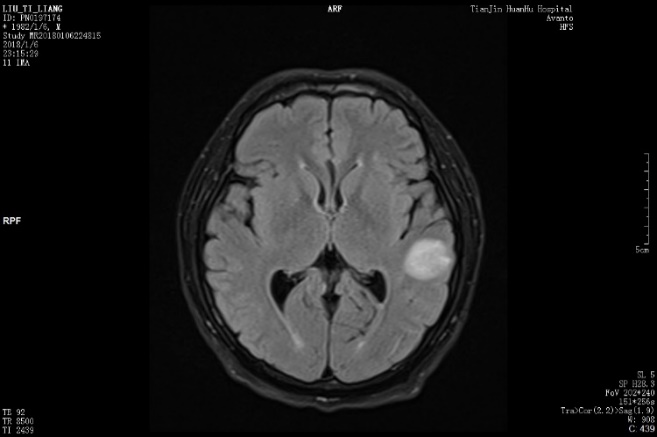

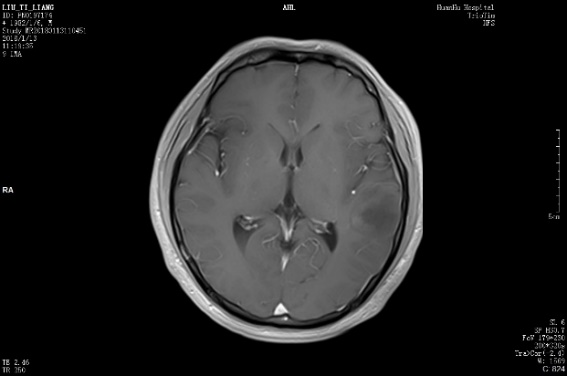


a b c

Figure S5: case 5, male, 37 years old, alalia for 1 week, MRI: abnormal signals in left temporal lobe.


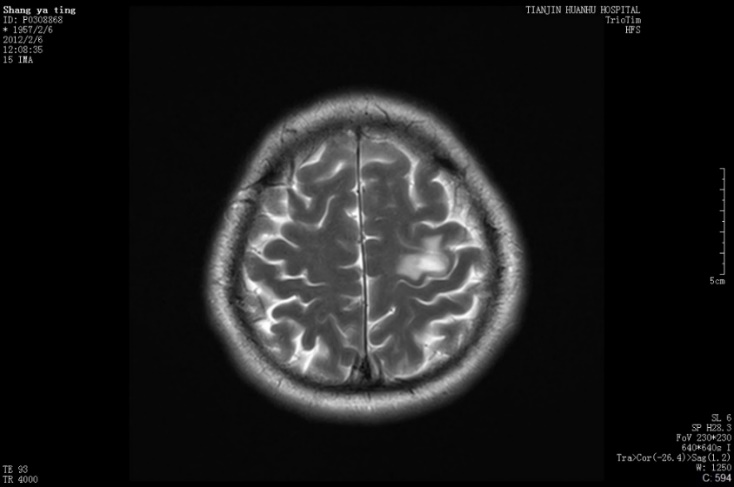

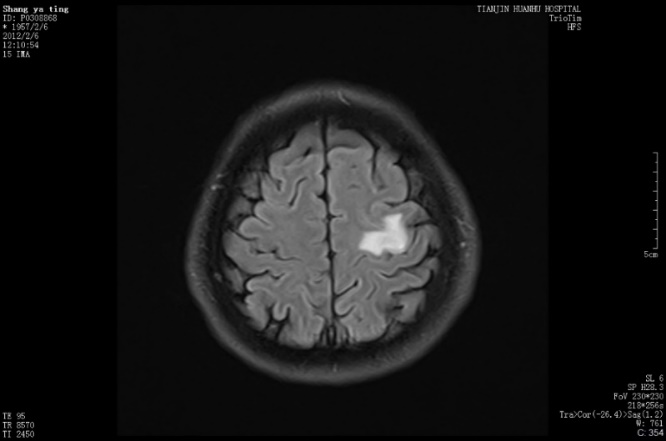

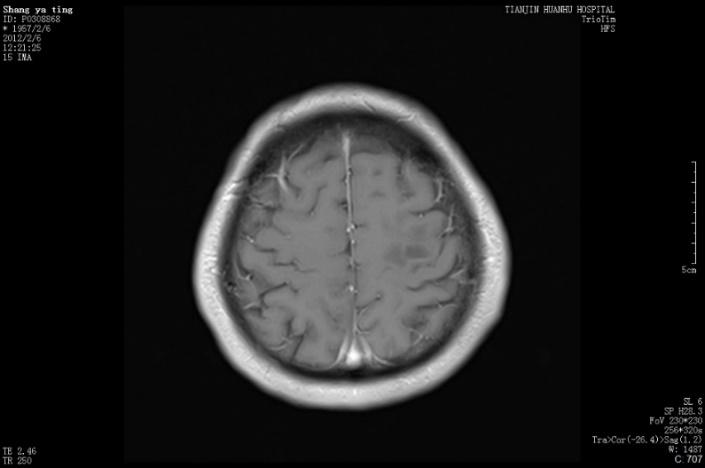


a b c

Figure S6: case 6, male ,55 years old, weakness of right limb for 14 days. MRI: abnormal signals in left frontal lobe.


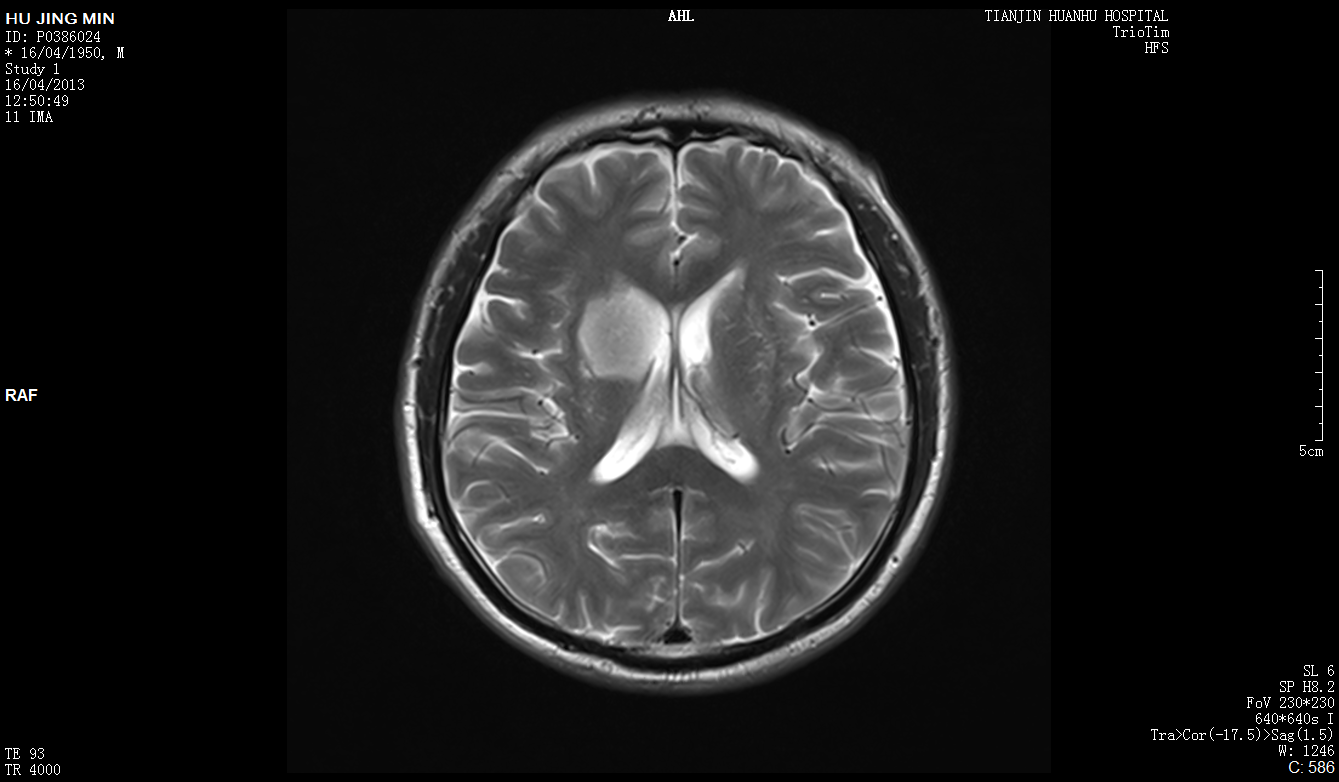

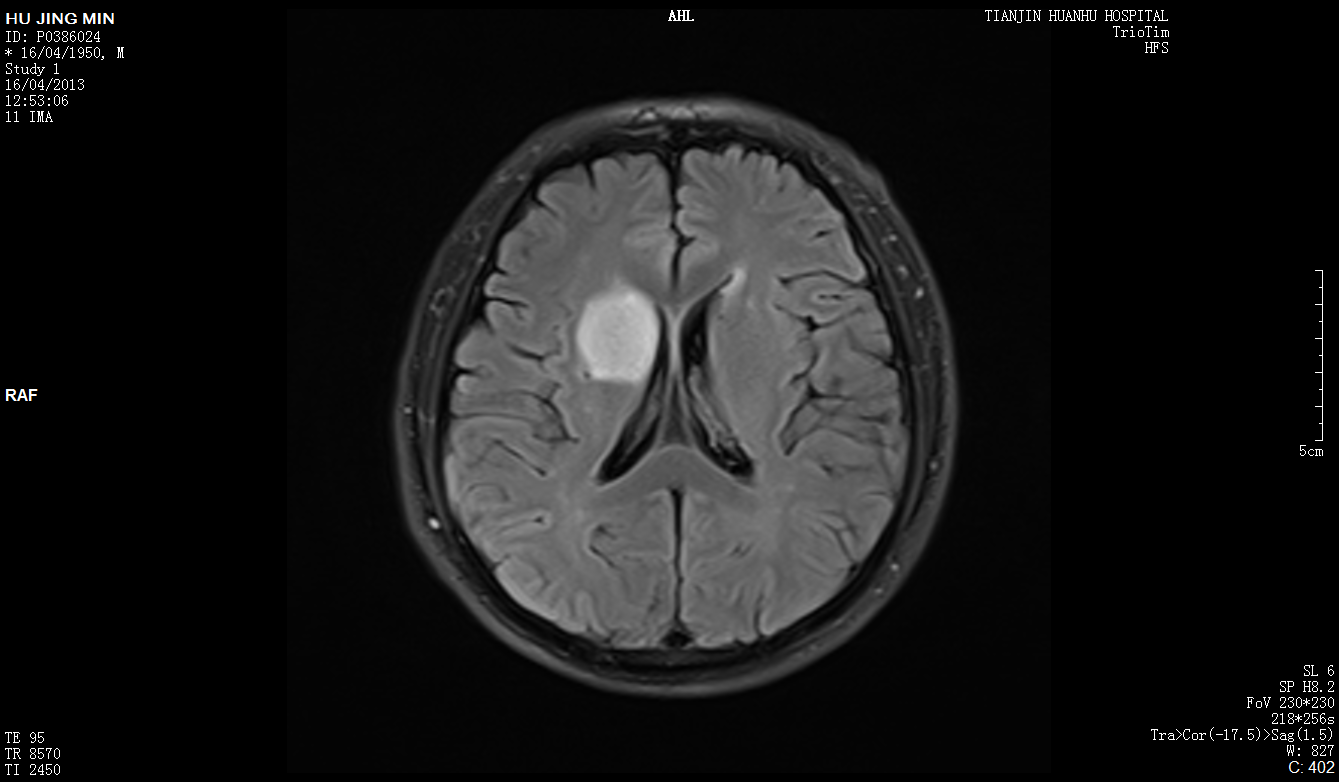

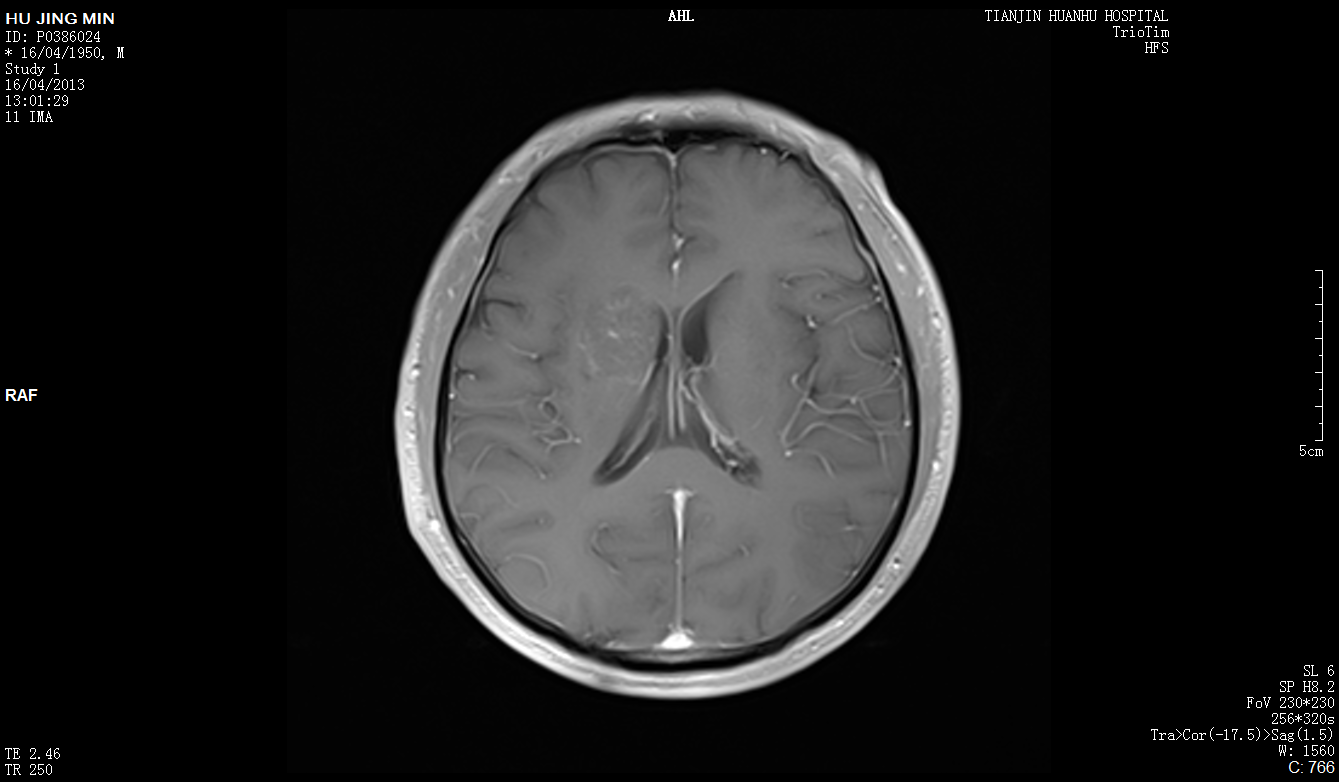


a b c

Figure S7: case 7, male, 62 years old. Headache and dizziness for 1 week. MRI: abnormal signals in right basal ganglia.


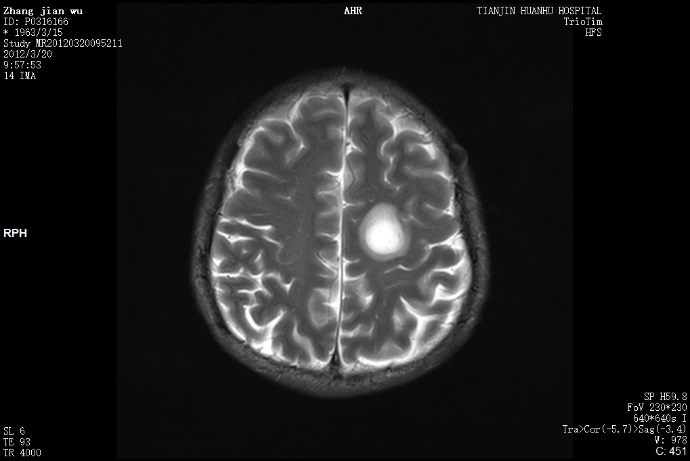

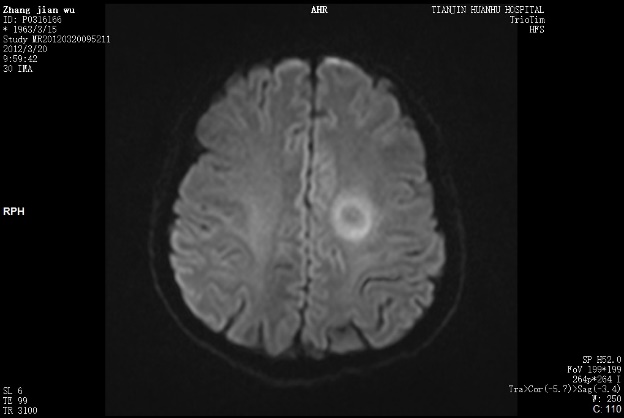

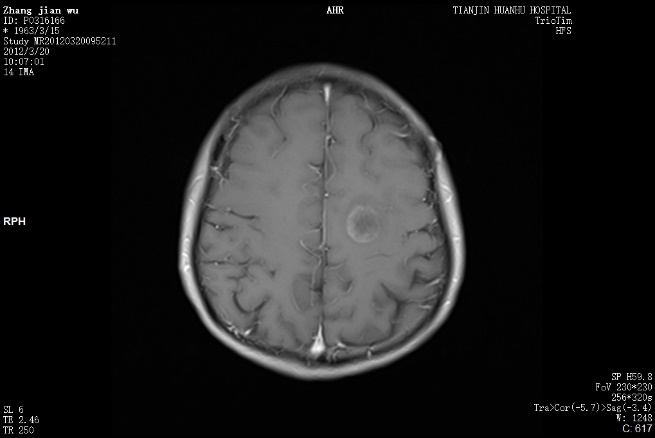


a b c

Figure S8: case 9. male, 49 years old, weakness of right limbs for 3 days. MRI: abnormal signals in left centrum semiovale.


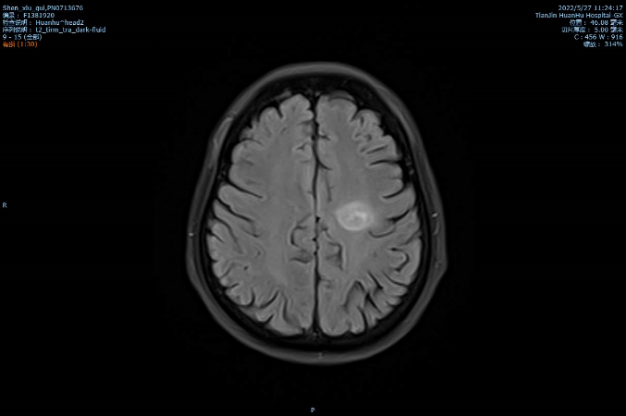

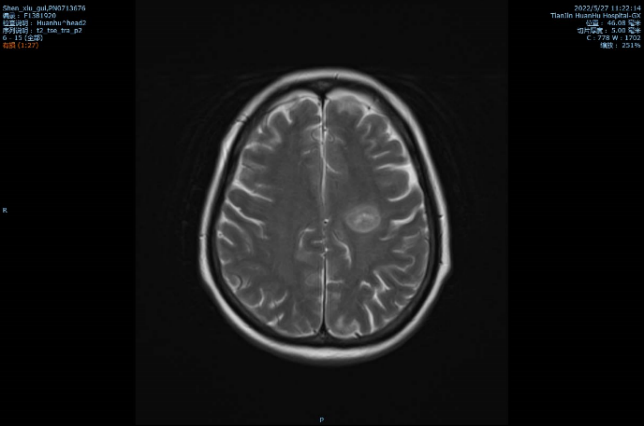

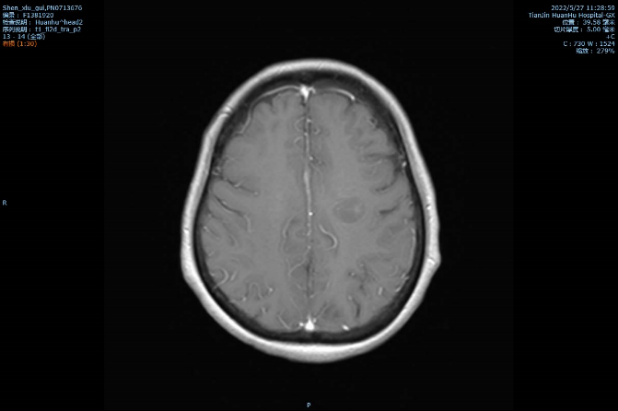


a b c

Figure S9: case 10, male ,50 years old, numbness and weakness of right limbs for 14 days. MRI: abnormal signals in left centrum semiovale.


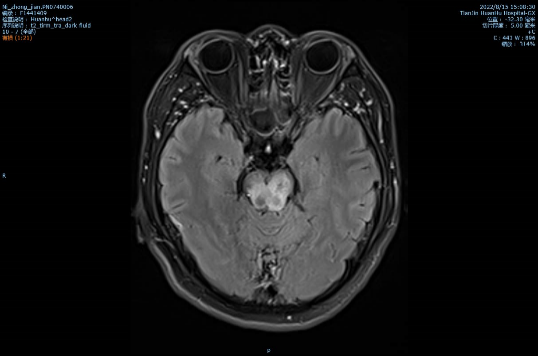

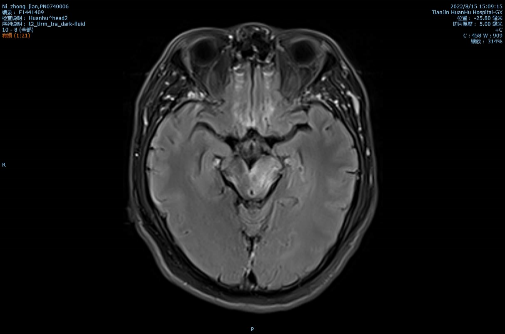

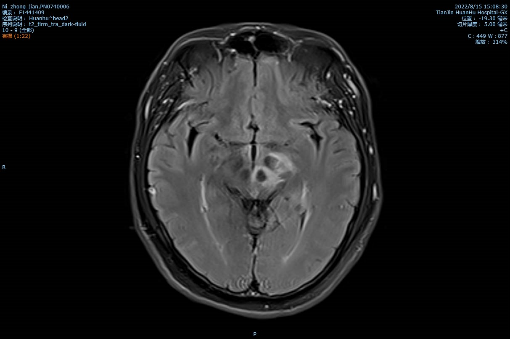

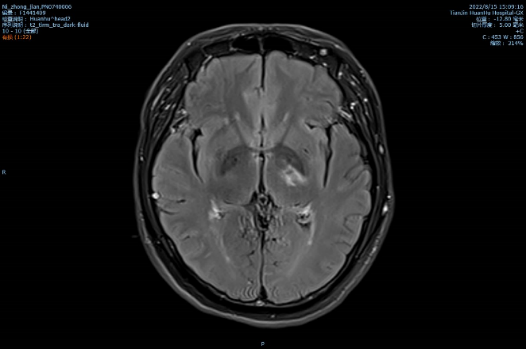


a b c d


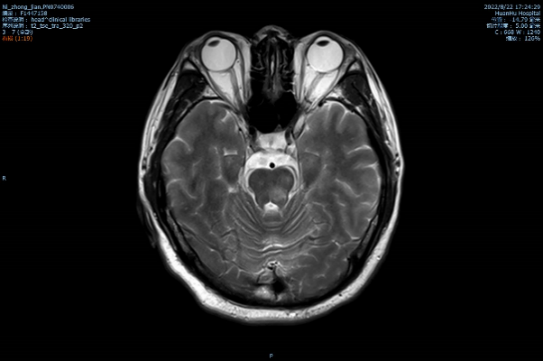

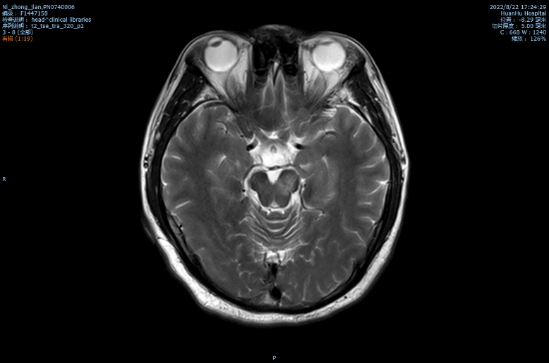

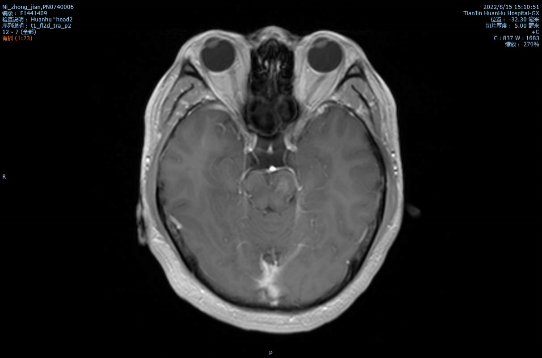

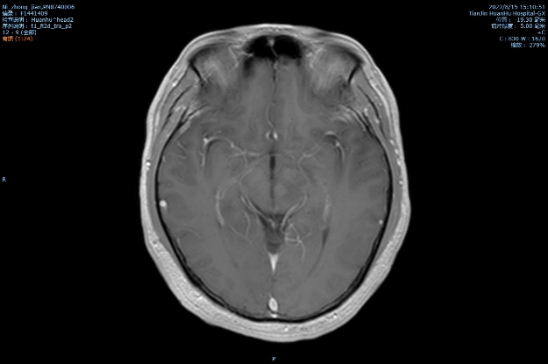


e f g h


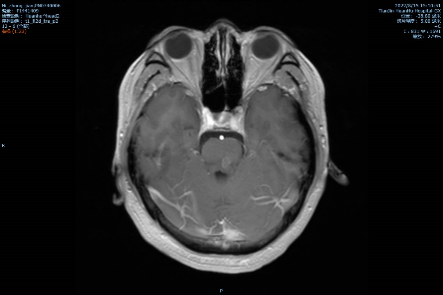

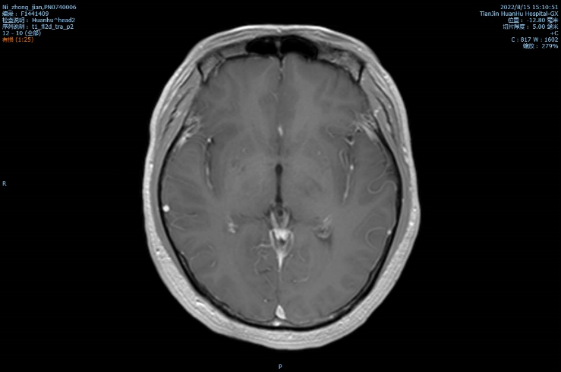


i j

Figure S10: case 11, female, 34 years old, alalia for 1 week. MRI: Abnormal signals in pons, midbrain and left thalamus.


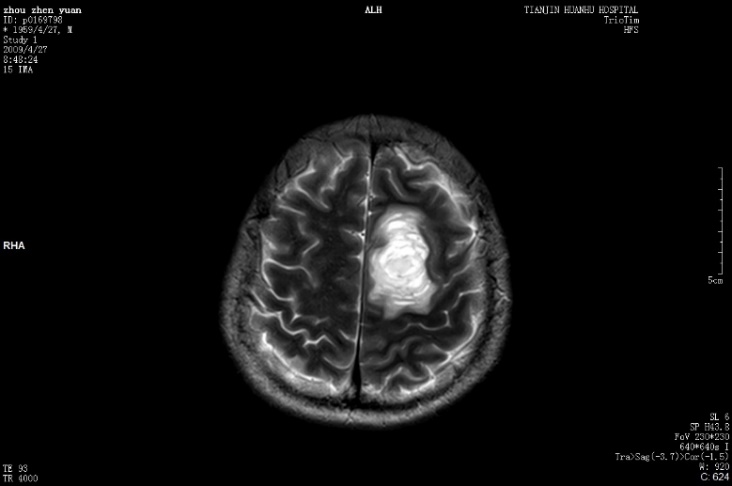

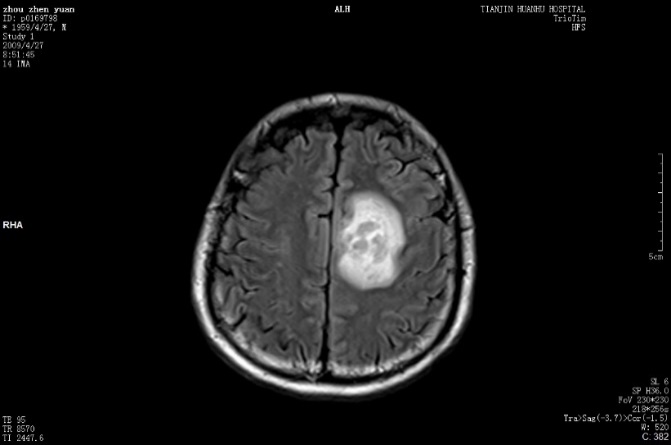

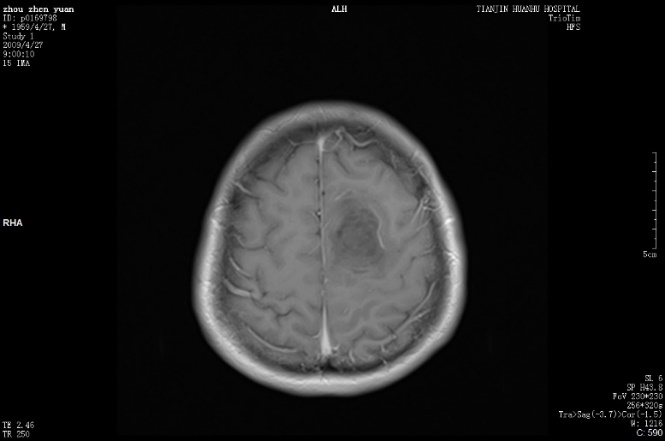


a b c

Figure S11: case 12，male, 29 years old, 10 days of paroxysmal convulsion and 10 days of dizziness. MRI: abnormal signals in left frontal and parietal lobe.
